# Correction to “MZT2A Promotes NSCLC Viability and Invasion by Increasing Akt Phosphorylation via the MOZART2 Domain”

**DOI:** 10.1111/cas.70271

**Published:** 2025-11-18

Wang H, Jiang X, Cheng Y, et al. MZT2A promotes NSCLC viability and invasion by increasing Akt phosphorylation via the MOZART2 domain. *Cancer Sci*. 2021;112:2210–2222. https://doi.org/10.1111/cas.14900

In the above article, Figure 3E is incorrect. The correct image is shown below:

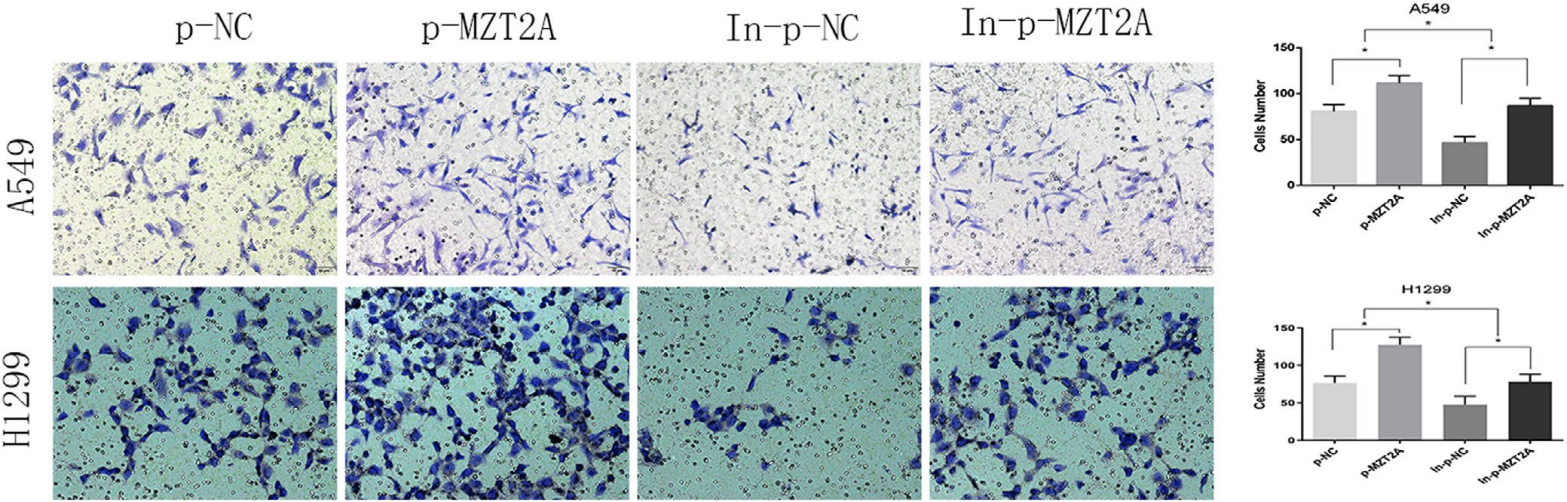

We apologize for this error.

